# Oral findings in patients with cartilage-hair hypoplasia - cross-sectional observational study

**DOI:** 10.1186/s13023-023-02758-7

**Published:** 2023-06-12

**Authors:** Heidi Arponen, Svetlana Vakkilainen, Jaana Rautava, Outi Mäkitie

**Affiliations:** 1 Department of Oral and Maxillofacial Diseases, University of Helsinki, and Helsinki University Hospital Head and Neck Center, Helsinki, Finland; 2 Children’s Hospital, Pediatric Research Center, University of Helsinki, and Helsinki University Hospital, Helsinki, Finland; 3 City of Espoo, Social and Health Services, Espoo, Finland; 4 Children’s Hospital, Helsinki University Hospital, Helsinki, Finland; 5 Department of Oral and Maxillofacial Diseases, HUS Head and Neck Center, Department of Pathology, HUSLAB Diagnostics, Helsinki, Finland; 6 Institute of Dentistry, University of Turku, Turku, Finland; 7 Research Program for Clinical and Molecular Metabolism, Faculty of Medicine, University of Helsinki, Helsinki, Finland; 8 Folkhalsan Research Center, Helsinki, Finland; 9 Department of Molecular Medicine and Surgery, Karolinska Institutet and Clinical Genetics, Karolinska University Hospital, Stockholm, Sweden

**Keywords:** Immunodeficiency, Inborn error of immunity, Cartilage-hair hypoplasia, Periodontitis, Oral mucosal lesion

## Abstract

**Background and objectives:**

Cartilage-hair hypoplasia (CHH) is a rare chondrodysplasia with associated primary immunodeficiency. The aim of this cross-sectional study was to examine oral health indicators in individuals with CHH.

**Methods:**

In total, 23 individuals with CHH, aged between 4.5 and 70 years, and 46 controls aged between 5 and 76 years were clinically examined for periodontal disease, presence of oral mucosal lesions, tooth decay, masticatory system function, and malocclusions. A chairside lateral flow immunoassay test of active-matrix metalloproteinase was obtained from all the adult participants with a permanent dentition. Laboratory signs of immunodeficiency were recorded for individuals with CHH.

**Results:**

Individuals with CHH and controls had similar prevalence of gingival bleeding on probing (median 6% vs. 4%). Oral fluid active-matrix metalloproteinase concentration was greater than 20 ng/ml in 45% of study subjects in both groups. However, deep periodontal pockets, 4 mm or deeper, were more common in individuals with CHH as compared to the controls (U = 282.5, p = 0.002). Similarly mucosal lesions were significantly more common in individuals with CHH (30% vs. 9%, OR = 0.223, 95%CI 0.057–0.867). The median sum of the number of decayed, missing due to caries, and filled teeth was nine for the individuals with CHH and four for controls. In the CHH cohort, 70% displayed an ideal sagittal occlusal relationship. Malocclusion and temporomandibular joint dysfunction prevalence were similar in both study groups.

**Conclusions:**

Individuals with CHH have more frequently deep periodontal pockets and oral mucosal lesions than general population controls. Routine intraoral examination by a dentist at regular intervals should be recommended to all individuals with CHH.

## Background

Cartilage-hair hypoplasia (CHH) is a rare autosomal recessive chondrodysplasia and an inborn error of immunity caused by a variant in the *RMRP* gene, which is the RNA component of mitochondrial RNA processing endoribonuclease [1]. The disorder affects bone growth plates and thereby leads to progressive growth failure. Clinical characteristics of CHH include short stature with short extremities, joint hypermobility, and hypotrichosis [2]. CHH is exceptionally prevalent among the North American Amish-population and in Finland [3], where the incidence is estimated to be 1:23 000 live births [2]. The spectrum of immunodeficiency in CHH is wide, ranging from asymptomatic to severe combined immunodeficiency [3–5]. The pathogenesis of immunodeficiency in CHH is incompletely understood, but involves abnormalities in cell cycle, telomere biology and ribosomal function [3]. In addition to decreased lymphocyte count and function, anemia and neutropenia can occur [3]. Consequently, individuals with CHH have an increased risk of infections and malignancies, particularly lymphoma and basal cell carcinoma [6, 7].

The most common oral diseases in the general population are periodontal disease and tooth decay, also known as caries [8]. Periodontitis, a chronic inflammatory disease, is triggered by bacterial micro-organisms resulting in immune-mediated destruction of tooth-supporting tissues [9]. An impaired immune status has been shown to impact oral bacterial profile and thereby increase the prevalence of periodontitis and caries, as well as the risk for oral colonization with *Candida* spp [10–12]. Matrix metalloproteinase-8 (MMP-8), expressed by neutrophils and macrophages, is a major destructive collagenase involved in periodontitis and caries lesions [9, 13]. Periodontitis in turn is considered a risk factor for some cardiovascular, respiratory, endocrine, musculoskeletal, and reproductive system related diseases [9]. In this cross-sectional observational study, we explored an association between CHH and oral health parameters. We hypothesized that immunodeficiency in individuals with CHH may lead to impaired oral health as compared with the general population. No previous study has investigated oral health in individuals with CHH. To date, only one report on the occlusal characteristics of 24 CHH patients aged from infancy to 21 years has been published [14].

## Patients and methods

The study protocol was approved by the Research Ethics Committee of the Hospital District of Helsinki and Uusimaa (HUS836/2018). In 2020 we invited all the 112 patients with CHH from the Finnish Chondrodysplasia registry to participate in the study. In total, 32 individuals with CHH consented. This study coincided with the global SARS-CoV-2 pandemic that hindered the participation of all the willing candidates, and impeded recruitment of age-matched population controls as intended. Eventually, 23 individuals with CHH were able to attend the clinical examination between March 2020 and March 2022. Of them, three were children aged between 4.5 and 11.3 years, in a mixed dentition phase, and 20 were adults with a permanent dentition (Table 1). Informed consent was obtained from all the participants or their legal guardians.

**Table 1:** Study population characteristics and clinical findings (dichotomous and categorical variables)

	Individuals with CHH n = 23	Controls n = 46	Difference between groups p value
Gender (male/female)	9/14 (39/61%)	17/29 (37/63%)	0.861^#^
Age mean (range) in years	40(5–70)	36(5–76)	0.321^##^
Children’s CHH growth percentile* < 10 10–25 50	1 1 1		
Adult height (cm)			
< 115	4		
115–130	9		
> 130	6	40	
Unknown	1		
Smoking (yes)	4 (17%)	3 (11%)	0.159^#^
Oral mucosal lesions (yes)	7 (30%)	4 (9%)	0.036^#^
TMD (yes)	4 (17%)	7 (15%)	0.809^#^
Oral fluid aMMP-8 > 20 ng/ml**	9/20 (45%)	18/40 (45%)	1.0^#^
Decay in deciduous dentition	1/3	0/6	
Profile convex normal concave	9 13 0	27 19 0	
Angle classification of occlusion I II III	16 7 0	31 14 1	0.709^#^
Overjet median (range) in mm	3(-3.5–8)	3(0–11)	
Overbite median(range) in mm	2(-2–6)	2(-3–8)	

CHH Cartilage-hair-hypoplasia

Controls Individuals without CHH

* Reference values percentile for height vs. age: Mäkitie et al.[47]

TMD Temporomandibular joint dysfunction

** Periosafe® test result positive by visual estimation in individuals with permanent dentition

# Chi-Square test

## Mann-Whitney U-test

The eventual population control group consisted of 46 volunteers recruited from a public health care clinic serving the entire local population. Of them, six were children aged between 5.0 and 11.6 years, corresponding to the age range of the patient group, and 40 were a random sample of adult patients (75%) and staff members (25%) of Espoo Municipality Dental Clinic.

Primary outcome measures were periodontal disease, presence of oral mucosal lesions, caries, and malocclusion. As a reference for evaluation of the power of the study, we used the reported average 36% prevalence of Finnish adults with no periodontal pocketing (deepest probing pocket depth less than 4 mm) and oral mucosal lesion prevalence of 10.5% [15, 16]. With 80% power (alpha 0.05) this study sample size would be able to reliably detect deepened periodontal pocketing incidence of 79% or higher in the patient group and oral mucosal lesion incidence of 50% or higher in the patient group.

### Clinical and radiological examination

Dentist (HA) carried out all the clinical examinations in a systematic manner as recommended by WHO Oral Health Surveys [17]. Full mouth visual examination included inspection of the periodontal tissues, oral mucosa, dentition, masticatory system, and occlusion. Mucosal changes were recorded and photographed. The photographs were analysed with a specialist in oral pathology to confirm the clinical diagnosis and a possible need for biopsy. Tooth-based recordings included gingival bleeding on probing (BOP), the depth of gingival pockets, and the status of all teeth. Gingival bleeding was scored as absent or present, and the number of sites where bleeding was present was recorded. Based on the measurement, bleeding on probing index was applied to numerically record the extent of gingival bleeding caused by inflammation [18]. Community periodontal index (CPI) classification was determined based on probing pocket depth and presence of dental calculus according to WHO-recommendation [19]. Pocket depth was measured with a periodontal probe (WHO/CPITN type E probe ball-ended probe) at six sites on each tooth. Community periodontal index value ranges from 0, indicating healthy periodontium, to 4, indicating severe periodontitis with pockets of 6 mm and deeper [19]. All teeth surfaces were inspected, and the dental status of each tooth was recorded as sound and untreated, filled, decayed (lesions reaching dentine, coronal and root caries), or extracted due to decay to establish dmft/DMFT (decayed, missing, filled primary/permanent teeth) index. Patient history of pain and functional limitations related to temporomandibular joint dysfunction were recorded, as well as joint tenderness, joint sounds (clicking and crepitation), range of motion, and deviations according to previous recommendation for diagnostic criteria for temporomandibular joint dysfunction [20]. Intra-oral occlusal characteristics were documented as overjet (mm), overbite (mm), and right/left first permanent molar anteroposterior relationship according to the Angle classification (Class I/II/III) [21]. When first permanent molars were unerupted or had been extracted, anteroposterior canine relationship was evaluated.

A chairside lateral flow immunoassay test (PerioSafe®, Dentognostics GmbH, Jena, Germany) was obtained from all adult participants with a permanent dentition to evaluate oral inflammatory burden and severity of periodontal disease by detecting oral fluid biomarker of active matrix metalloproteinase (aMMP-8) [22, 23]. The test was performed from a mouth rinse, following the manufacturer’s instructions, leading to either a positive or negative visual test result. PerioSafe® point-of-care test has been successfully validated to differentiate periodontal health and disease in individuals with permanent dentition [22]. Patients were interviewed for subjective problems related to oral health and smoking status. Repeating the clinical examination would not have been feasible.

Panoramic radiographs, obtained to examine dental development and eruption of permanent teeth in mixed dentition phase, were assessed for dental age in two individuals with CHH; one aged 9.8 years and the other 10.1 years at the time of radiographing. Reliability of the radiographic analysis was verified by the same examiner (HA) repeating the assessment. Dental development findings were evaluated against the age medians given for Finnish children by Haavikko [24].

Clinical and laboratory characteristics of the majority of the patients have been reported in our previous studies [6, 7]. Data collected and analyzed in the current report included laboratory signs of immunodeficiency (lymphopenia, neutropenia, hypogammaglobulinemia).

### Statistical analyses

Shapiro-Wilk’s test of normality was applied to examine distribution of the data. Mann-Whitney U-test was used to compare the distribution of bleeding on probing, community periodontal index, and number of decayed, missing, filled primary/permanent teeth across the patient and control groups. Chi-square (χ^2^) test was used to analyze group differences of mucosal lesions, MMP-8 activity, smoking status, temporomandibular joint dysfunction symptoms, and Angle classification of the occlusion in the patient and control groups. Relationship between bleeding on probing, community periodontal index, and active MMP-8 were examined with Spearman’s rank correlation test. Statistical analyses were performed with IBM SPSS Statistics software (version 27). Significance was considered for p < 0.05 (2-sided). Continuous data are presented as medians and interquartile ranges (IQR) [25th–75th percentile].

## Results

Table 1 outlines the study cohort characteristics. Mean age of the controls was four years less than that of individuals with CHH, whereas the eldest CHH patient was six years younger than the eldest control. However, the age difference between the groups was not statistically significant. Two patients and one control had been diagnosed with sleep apnea. Neutropenia had previously been documented in one patient (4%) [3], who presented with a positive oral fluid aMMP-8 test (aMMP-8 level > 20 ng/ml) in clinical examination but no generalized gingival bleeding on probing or deepened periodontal pockets (community periodontal index value 1). In total, 12 patients (52%) had been diagnosed with lymphopenia. Anemia had been detected in one of the three children with CHH (reference Hb < 112 g/l for under 8-year-olds and Hb < 116 g/l for under 12-year-olds). One control had received a heart transplant and was receiving immunosuppressive therapy. She exhibited a negative aMMP-8 oral fluid test result and no deepened periodontal pockets. One control was currently in orthodontic treatment with fixed appliances and presented with a positive aMMP-8 test. One patient and one control had orthognathic surgery planned to correct severe malocclusion. One patient and three controls had a full deciduous dentition. Two patients and three controls were in a mixed dentition phase. Dental age of the individuals with CHH and mixed dentition was in line with chronological age. Of the adults with permanent dentition, seven individuals with CHH (35%) and 18 controls (45%) had missing teeth (excluding wisdom’s teeth).

**Fig. 1:**
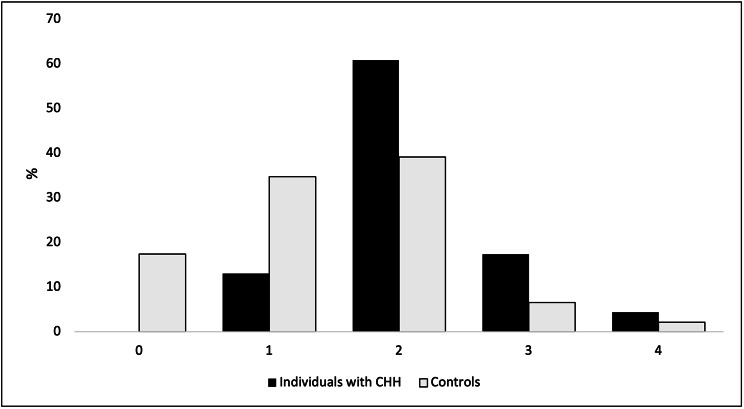
Percent distribution of community periodontal index scores by study group

Smoking was equally common among individuals with CHH and controls χ^2^ (1,n = 69) = 1.987 (OR = 0.331, 95%CI 0.067–1.627) (Table 1). Similarly, bleeding on probing percentage and oral fluid aMMP-8 test results did not differ significantly between individuals with CHH and controls (U = 383.5 and χ^2^ (1,n = 60) = 0.0 (OR = 1.0, 95%CI 0.340–2.942) respectively) (Table 2). In contrast, community periodontal index score values were significantly different between the groups (U = 282.5, p = 0.002). Deep periodontal pockets (community periodontal index 3 and/or 4), as a symptom of periodontitis, were more frequent in patients (Fig. 1). Community periodontal index score and bleeding on probing score correlated positively with MMP-8 activity (r_s_ =0.414, p = 0.001 and r_s_ =0.298, p = 0.021 respectively), and with each other (r_s_ =0.463, p = 0.00). In none of the CHH patients but in one control the gingival bleeding was considered generalized as defined by a bleeding on probing percentage of 30% of sites or higher [18]. In line with the finding of generalized bleeding on probing, the community periodontal index score and aMMP-8 test of the individual were also indicative of periodontitis.

**Table 2:** Oral health indices

	Patients with CHH					Controls					Difference between groups
	n	Min	Max	Median (IQR)	Missing (n)	n	Min	Max	Median (IQR)	Missing (n)	p-value
BOP	23	0	22	6(6)	0	44	0	31	4(6)	2	0.104
CPI	22	1	4	2(1)	1	46	0	4	2(1)	0	0.002^#^
0	0					8					
1	3					16					
2	14					18					
3	4					3					
4	1					1					
dmft/DMFT	22	1	24	9(12)	0	44	0	19	4(8)	0	0.053

BOP Bleeding on probing -index percentage

CPI Community periodontal index. Severity and degree of periodontal diseases in an individual, ranging from 0 (healthy, inflammation-free gingiva and periodontium) to 4 (most severe form of periodontitis with loss of function of the teeth)

dmft/DMFT Number of Decayed, Missing due to decay, or Filled Teeth, out of 20 deciduous or 28 permanent teeth. DMFT index score minimum value is 0 and maximum 28 in an adult

# Difference with controls was statistically significant (Mann Whitney U test 2-sided)

**Fig. 2:**
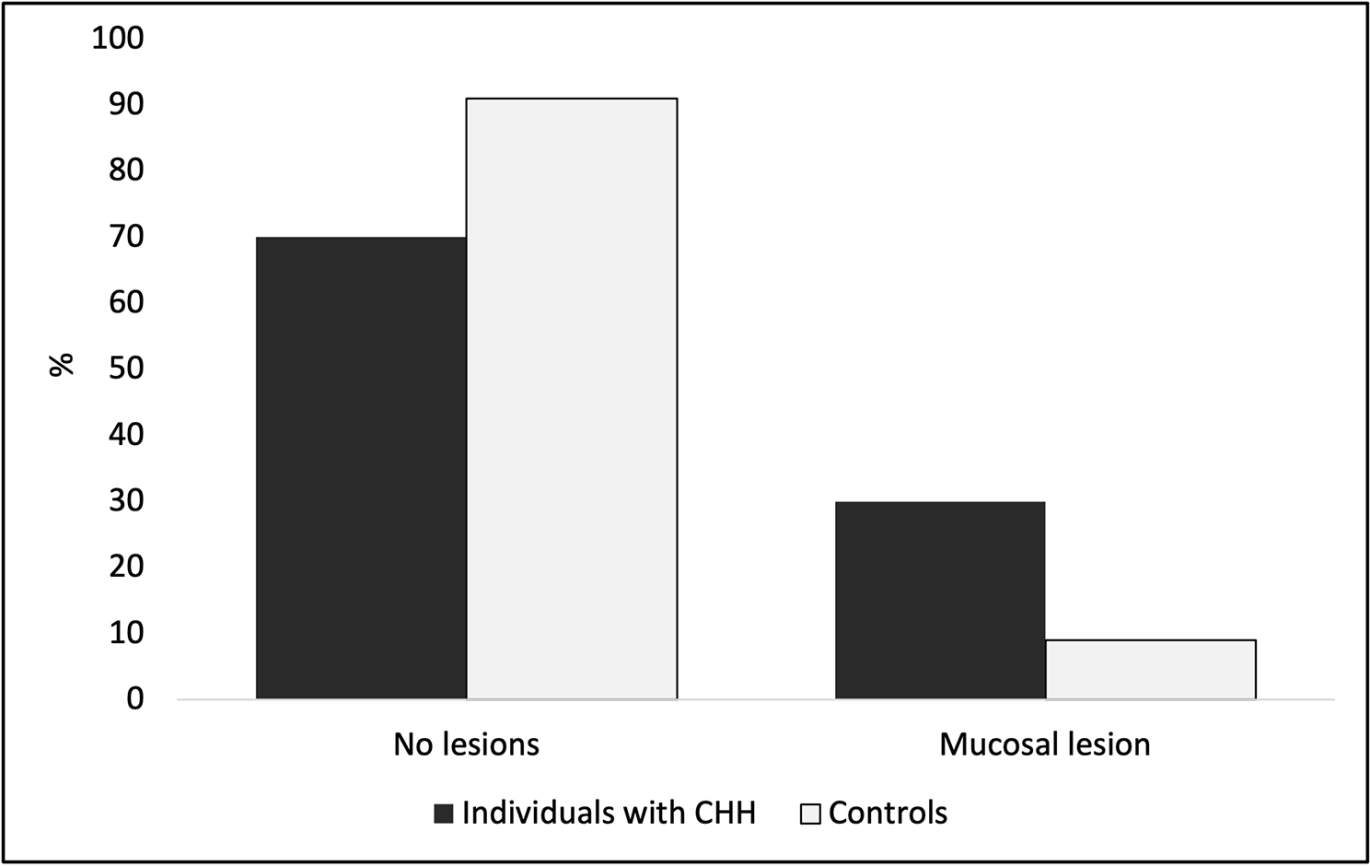
Percent distribution of oral mucosal lesion observation by study group

**Fig. 3:**
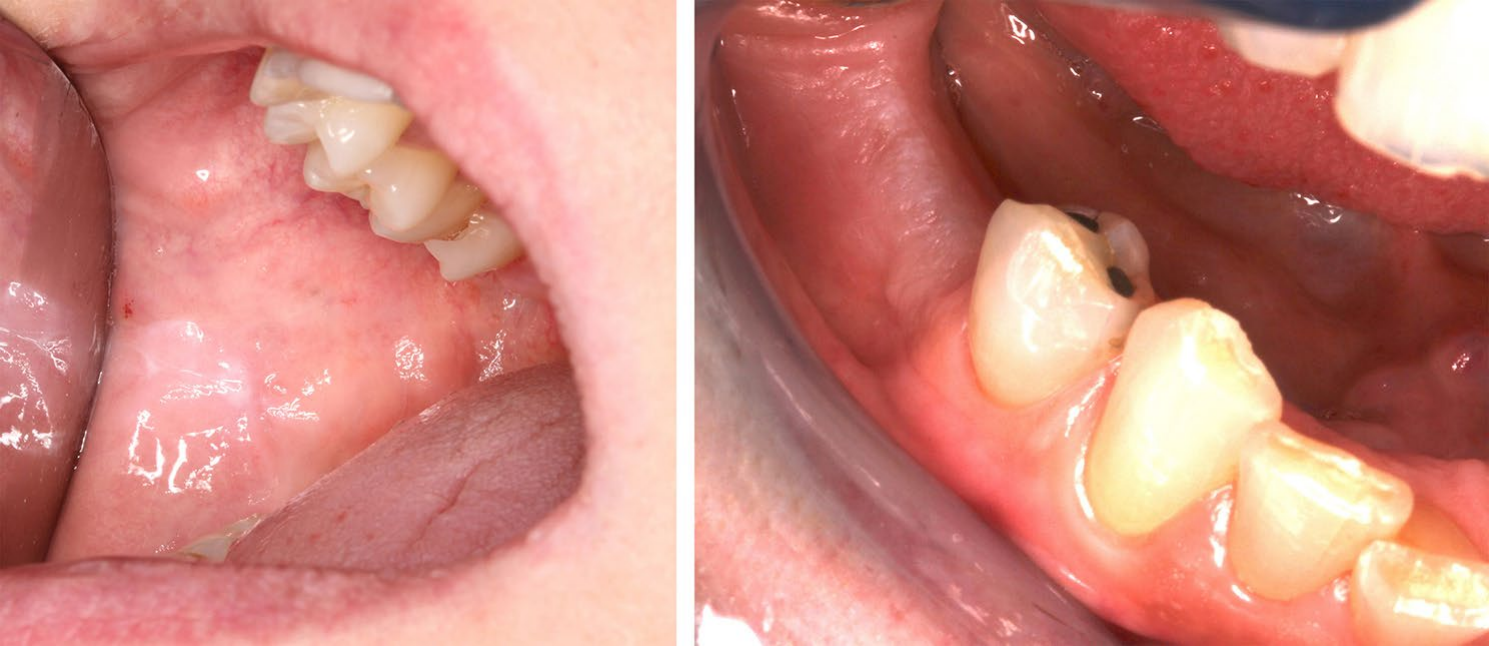
Reactive hyperkeratinization on buccal mucosa and reactive hyperkeratinization on alveolar mucosa

In this cohort, mucosal lesions were a more frequent finding in individuals with CHH as compared to controls χ^2^(1, n = 68) = 5.211, p = 0.036 (OR = 0.223, 95%CI 0.057–0.867) (Fig. 2). Seven patients and one control displayed reactive hyperkeratinization (oral frictional hyperkeratosis) either on the buccal mucosa along the occlusal line or on edentulous areas of the crista (Fig. 3). One patient with poor oral hygiene had bilateral asymptomatic lichenoid reaction on the sublingual mucosa. The patient was using coconut oil as a mouth rinse twice a day and no toothpaste. He had suffered from xerostomia and a swollen submandibular salivary gland for several years. One control had candidiasis of the palate, one displayed a food allergy-related mucosal reaction of the lips, and one control child exhibited aphthous mouth ulcers.

The decayed, missing, filled primary/permanent teeth score was higher in the patient group, but the difference did not reach statistical significance (U = 378.0) (Table 2). The average decayed, missing, filled primary/permanent teeth score of individuals under the age of 35 years was five in the patient group and three in the control group. The decayed, missing, filled primary/permanent teeth score of adults divided into two age groups, as recommended by WHO [17], of 35–44 and 65–74 years, was nine vs. seven and 15 vs. 11 for the patients and controls, respectively. A child with CHH and anemia had one treated caries lesion, whereas another child with CHH and no anemia had caries in six out of 20 deciduous teeth. A child with CHH and no anemia had intact teeth.

Temporomandibular joint dysfunction was reported by four individuals with CHH (17%) and seven controls (15%) (χ^2^ (1, n = 69) = 0.058, OR = 1.197, 95%CI 0.279–5.132) (Table 1). Of them, the four patients and three controls experienced bruxism and myalgia. Four controls described temporomandibular joint symptoms of a disc displacement and/or subluxation of the condyle. Joint sounds were reported by two patients. None of the study subjects had limited range of jaw movement or deviation of movement.

Normal overjet, defined as a 0.5 to 5.5 mm distance between the incisor teeth [25], was exhibited by all but two of the patients (Table 1). All but one patient exhibited a positive overbite. All the controls displayed a positive overjet and all but two a positive overbite. Crowding was present in three patients (13%) and a cross-bite in five patients (22%) of whom four displayed lateral cross-bite and one anterior cross bite. Deep bite was present in one patient (4%), anterior open bite in one (4%), and scissors bite in one (4%). The sagittal occlusal relationship, as defined by Angle classification, did not differ significantly between the patients and controls χ^2^ (3,n = 69) = 1.386 (95%CI 0.768–0.785).

## Discussion

This is the first study to report oral findings on individuals with CHH, a severe skeletal dysplasia with immune deficiency. In line with our expectations, we found that individuals with CHH have more frequently severe periodontitis and oral mucosal lesions than population controls.

Oral cavity hosts diverse microbiota which includes bacteria, viruses, and fungi that contribute to metabolic, physiological, and immunological functions [26]. A dysbiotic microbiota can induce destruction of the periodontal tissue by a dysregulated host inflammatory immune response and disturbe the neutral environment of the oral cavity, predisposing to demineralization of tooth enamel [26]. Chronic inflammatory conditions may increase the risk of periodontal disease [27, 28]. In contrast, immunosuppressive therapy following renal transplantation induces no change in susceptibility to destructive periodontal disease or in the decayed, missing, filled primary/permanent teeth index [29]. Individuals with CHH and symptomatic immunodeficiency typically suffer from recurrent respiratory tract infections [3]. Immunodeficiency could influence the susceptibility of individuals with CHH to periodontal disease.

Periodontitis is one of the most common human inflammatory diseases. Overall, 8 − 10% of the general population presents with periodontal disease [30]. Our control group is a representative sample of the general population regarding the prevalence of periodontitis (9%). The prevalence of periodontitis was higher in individuals with CHH. In our cohort, 22% of the patients exhibited deepened periodontal pockets (community periodontal index 3 or 4). Previous investigations have shown that concentration of MMP-8 in oral fluid correlates with the degree of inflammation in a patient with periodontal disease and with dental decay [13, 22]. This accords with our observations.

Bleeding on probing typically precedes development of gingivitis and periodontitis [31]. Notably, bleeding on probing percentage and positive aMMP-8 point-of-care test prevalence, which are indicative of inflammation, were similar in patients and controls of our cohort. Previous investigation has shown that the diagnostic accuracy of aMMP-8 point-of-care immunoassay may be compromised in individuals with additional inflammatory conditions [32], which might explain our finding. Of the five patients with clinically detected deepened periodontal pockets, bleeding on probing percentage ranged between 2.8% and 22%, and aMMP-8 test was positive in four. At group level, as expected, those with more bleeding on probing and deeper gingival pockets had more often MMP-8 activity in oral fluids. Impaired T cell function in individuals with CHH [6] seems not to be reflected to aMMP-8 secretion to oral fluids.

Health behavior and hygiene habits impact development and progression of oral diseases [33]. Periodontitis increases the systemic inflammatory burden, as pro-inflammatory and tissue destruction mediators are released from the inflamed periodontal tissue to the circulatory system [33]. Therefore, maintaining good oral health is important in immunocompromised individuals with CHH.

In oral cavity, both mucosal and systemic immunity contribute to protection against infections and damage of the mucosa [34]. Of middle-aged Finnish population, 10.5% exhibit some mucosal changes [16]. White lesions of the oral cavity are relatively common incidental findings that have a variety of malignant or benign etiologies, including parafunctional habits, mechanical friction, contact reactions, as well as chemical-related changes [35]. Oral frictional hyperkeratosis is a benign asymptomatic white lesion of the oral mucosa caused by chronic trauma to the site [35]. Its prevalence in general population has been estimated at 5.5% [36]. Oral lichen planus is an idiopathic T lymphocyte mediated chronic inflammatory disorder that most often affects middle-aged adults [37]. Medications or contact allergens can cause lichenoid reactions [37]. Immunosuppression might be assumed to emphasize the inflammatory process in mucosal lesions [38]. This assumption would be supported by our finding of a high prevalence (30%) of oral mucosal changes in individuals with CHH.

Decayed, missing, filled primary/permanent teeth score is globally the most important index for assessing the status of dental health [39]. The decayed, missing, filled primary/permanent teeth score value increases with age [40, 41]. This trend is confirmed in our study. In our cohort, the number of decayed, missing due to caries, and/or filled teeth was similar in patients and in controls, despite the eldest control being 6 years senior to the eldest patient. This finding suggests better dental health of the controls compared to age-matched individuals with CHH. In addition to the individual’s hygiene and dietary habits, medication and systemic diseases may affect saliva secretion and thus are also important in the development of dental decay [42].

Loose joints are an inherent feature of CHH [4]. Joint hypermobility in turn is a risk factor for temporomandibular disorders [43]. In our study, however, temporomandibular joint dysfunction was not more prevalent in individuals with CHH as compared to the controls. We have previously shown that the jaws of individuals with CHH are shorter than the average of age-matched general population, but proportionate to each other [44]. In the current study, 70% of the patients displayed an ideal occlusion and the malocclusion prevalence did not differ significantly between the patient and control groups. Distal bite was the most common malocclusion of the patients with CHH (30%) followed by lateral crossbite (22%). Previous studies report a 18% prevalence of lateral crossbite in Finnish adult population [45].

Finally, a number of important limitations need to be considered. First is the possible selection bias of both the patients and controls, as individuals conscious of their oral health would be expected to enroll for an examination. The control group included dental clinic staff members, who would be likely to maintain good oral hygiene practices thereby influencing bleeding on probing, community periodontal index, and decayed, missing, filled primary/permanent teeth score. Assessment of oral hygiene practices and nutrition was not included in the study. Sensitivity of the clinical oral examination in detecting dysplastic and malignant oral lesions has been found to be good, whereas the specificity is poor [46] and must be interpreted with caution. Histological diagnosis is needed to verify the clinical diagnosis of mucosal lesions for better specificity. The oral mucosal lesions in this study were examined by a specialist in oral mucosal diseases and no need for biopsy was deemed. In addition, with a small sample size, findings might not be transferable to the whole CHH population.

## Conclusions

The importance and originality of this study is that this is the first report on oral health of individuals with CHH. Due to the prevalent occurrence of deep periodontal pockets and mucosal lesions, routine intraoral examination at regular intervals should be recommended to all patients. Information gained from this study can be used to develop targeted interventions aimed at improving health of individuals with CHH.

## Data Availability

The datasets used and/or analysed during the current study are included in this published article. All data generated are available from the corresponding author on reasonable request.

## Abreviations

BOP: Bleeding on probing
CHH: Cartilage-hair hypoplasia
dmft/DMFT: decayed, missing, filled primary/permanent teeth
MMP: Matrix metalloproteinase
TMD: temporomandibular joint dysfunction
